# Supplementary material for: Factors associated with mortality in patients hospitalized for COVID-19 admitted to a tertiary hospital in Lambayeque, Peru, during the first wave of the pandemic
Source: PLoS One. 2023 May 11;18(5):e0285133. doi: 10.1371/journal.pone.0285133 (PMC10174592; doi:10.1371/journal.pone.0285133)
Supplement: Attachment — Submitted filename: Comments for the authors.doc [file pone.0285133.s001.doc]

I am submitting my comments in reference to the manuscript “Factors associated with mortality in patients hospitalized for COVID-19 admitted to a tertiary hospital in Lambayeque, Peru”. After going through the manuscript I found.

1.) The data presented is very much relevant to the scientific community.
2.) The manuscript has many typing mistakes and errors in sentence formation eg. Line 160 Abbreviation is ARDS but its expansion is Severe Respiratory Distress Syndrome.
3.) Many others are there which cannot be elaborated here.

It is advised to get the manuscript checked by an expert for English correction and resubmit.
