# Supplementary material for: Factors associated with mortality in patients hospitalized for COVID-19 admitted to a tertiary hospital in Lambayeque, Peru, during the first wave of the pandemic
Source: PLoS One. 2023 May 11;18(5):e0285133. doi: 10.1371/journal.pone.0285133 (PMC10174592; doi:10.1371/journal.pone.0285133)
Supplement: Attachment — Submitted filename: renamed_f048c.docx [file pone.0285133.s002.docx]

Sugiero que el titulo incluya que se realizo el estudio en la primera ola para así diferenciarlo de futuros estudios que determinen las características halladas en la segunda, tercera y cuarta ola ya que características como la morbimortalidad podrían ser diferentes.
