# Supplementary material for: Factors associated with mortality in patients hospitalized for COVID-19 admitted to a tertiary hospital in Lambayeque, Peru, during the first wave of the pandemic
Source: PLoS One. 2023 May 11;18(5):e0285133. doi: 10.1371/journal.pone.0285133 (PMC10174592; doi:10.1371/journal.pone.0285133)
Supplement: Attachment — Submitted filename: Response to Reviewers.docx [file pone.0285133.s003.docx]

**LETTER TO REVIEWERS**

Thank you very much for your comments and recommendations that have improved the quality of the article. In the following paragraphs we will report on the lifting of comments and acceptance of suggestions.

1. Your ethics statement should only appear in the Methods section of your manuscript. If your ethics statement is written in any section besides the Methods, please delete it from any other section. [Please check Line 377-382]

**Our response:** It was corrected according to their recommendations.

2. Please remove your figures within your manuscript file, leaving only the individual TIFF/EPS image files. These will be automatically included in the reviewer’s PDF

**Our response:** It was corrected according to their recommendations.

3. We note that your updated Data Availability statement in the submission system says: "10.6084/m9.figshare.21664172". However, the Data Availability statement in your manuscript says: "The data are not publicly available due to their containing information that could compromise the privacy of research participants."

**Our response:** Access to coded data was modified and added.
